# “Nature Hath Fram’d Strange Fellows in Her Time”

**DOI:** 10.3201/eid1309.000000

**Published:** 2007-09

**Authors:** Polyxeni Potter

**Affiliations:** * Centers for Disease Control and Prevention, Atlanta, Georgia, USA

**Keywords:** Paolo Veronese, Paolo Caliari, Venice, Juno, Late Renaissance Painting, Juno Bestowing Her Gifts on Venice, Venice Receives from Juno the Doge’s Hat, Mannerism, Illusionism, Serenissima, coronavirus, corona, about the cover

**Figure:**
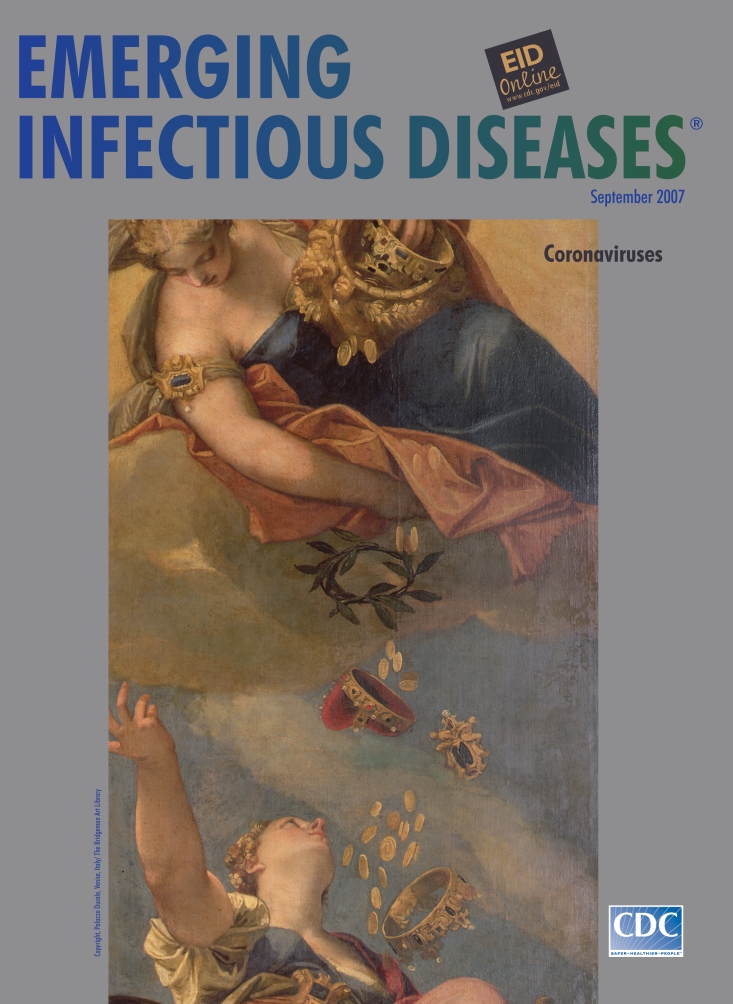
**Paolo Veronese (Paolo Caliari, 1528–1588). Venice Receives from Juno the Doge’s Hat, 1555 (detail).** Palazzo Ducale, Venice, Italy/The Bridgeman Art Library Nationality/copyright status: Italian/out of copyright

—William Shakespeare, The Merchant of Venice

Painters take the same license as poets and madmen, Paolo Veronese told the Inquisition Tribunal in Venice during an interrogation. Buffoons, drunkards, exotic creatures, and anachronisms in his Last Supper were placed there “so they might be of service because it seemed to me fitting ...” in creating the scene, not as irreverence (*1*). The dispute was resolved by changing the name of the painting to Supper in the House of Levi. Veronese was not interested in piety or historical accuracy. Large banquets were opportunities to create feasts for the eyes, monumental gatherings framed in architectural detail, bathed in sumptuous color.

The son of a stone mason known only as Gabriele, the painter adopted the name Caliari and later became known as Veronese from his birthplace. A precocious child entirely uninterested in stone cutting, he was quickly recognized for facility with the brush and was trained by local masters Antonio Badile and Giovanni Caroto. Then, according to chronicler Giorgio Vasari, architect and engineer Michele Sanmicheli took him under his wing and “treated him like a son” (*2*).

He painted his first works in Verona and Mantua, but when called to Venice on a commission, he remained there for the rest of his life, becoming a preeminent master of the late Renaissance, along with Titian and Tintoretto. In the Doge’s palace, the Church of San Sebastiano, the Villa Barbaro at Maser with the great architect Andrea Palladio, and churches and palaces all over the city, he extolled youth, beauty, and prodigious harvests in frescoes and oil paintings of enduring charm. A kind and amiable man, Veronese was well liked and appreciated, one of the first painters whose work was sought by collectors during his lifetime (*3*).

Early training in the mannerist style, which emphasized the decorative, was transformed by the styles of Venice, an innate sense of composition, and his genius as draftsman. He reveled in rich textures and patterns and captured luminescence in flesh and fabric, lace or wool. He was “the greatest colorist who ever lived,” wrote French critic Théophile Gautier, “greater than Titian, Rubens, or Rembrandt” because he created light without violent contrasts and maintained the strength of hue and shadow, which French master Eugène Delacroix (1798–1863) said, “We are always told is impossible” (*4*).

An expert illusionist, Veronese overcame the problems of applying linear perspective to the concave surfaces of church domes, overriding the architecture, simulating limitless space. With *sotto in su* techniques, he created foreshortened figures to be seen from below as floating above the viewer. He moved adventurously between secular and religious themes, incorporated classical and mythologic figures, crafted allegorical pageants, mingled the sacred with what some thought the profane.

Venice, la Serenissima or Most Serene Republic, and the myths surrounding her mercantile empire lent themselves to the theatrical, apotheotic exuberance of Veronese’s style. The city, described by Petrarch in 1364 as “rich in gold but richer in renown,” mythologized herself—Venetia, Queen of the Andriatic, at once pagan and medieval, her heritage not so much of classical Rome but the Byzantine East (*5*). He painted her effortless grandeur in gowns of gold brocade, seated on clouds, trumpeted by angels, showered with jewels from the gods.

Venice inspired generations of poets and writers from William Shakespeare and Lord Byron to Thomas Mann. And Veronese influenced the course of European art—in the 17th century through Rubens and Velazquez, in the 18th, through Giovanni Battista Tiepolo and others.

Juno, the Roman goddess bestowing gifts on Venice in Veronese’s brilliant allegory on this month’s cover, was none other than Greek goddess Hera, powerful wife of Zeus. In antiquity, her giving was legend, for havoc as well as gifts. She ruined foes but sanctioned marriage, her generosity even celebrated by Shakespeare, “Honour, riches, marriage-blessing, / Long continuance, and increasing, / Hourly joys be still upon you! / Juno sings her blessings on you” (*6*). In this, another of her less bellicose appearances, Juno rains gold and crowns on Venice, grooming her for greatness and prosperity. Afloat in sensuous color, she glances down at her. An olive branch, signifying honor, acknowledges a city “mighty in her resources but mightier in virtue” (*7*).

The extravagance of Juno’s gesture and its gracious acceptance bespeak the mythic greatness and splendor of Venice. Poetry and utopian texts, as well as the art of Veronese’s time, attributed this greatness in part to topography—though lapped by the waves, Venice maintained close ties with the northern mainland and amassed a land empire, the *terraferma* (dry land). Another link to greatness was harmonious interaction with nature and the cosmos. Venetian humanist Pietro Bembo proposed “ideal love” as key to this interaction. Likewise, Jacopo Sannazzaro in L’Arcadia (1500) attributed moral and spiritual perfection to human connection with the natural world and its rhythms (*7*).

La Serenissima succumbed in the late 1700s, becoming a *ville crépusculaire* (city like any other). Like the original Arcadia, she had existed largely in the imagination. Connection with nature, indispensable to the myth, survived the fall of the empire; poets, painters, and scientists still seek it in Venice and elsewhere.

Bejeweled crowns from above, royal coronas, seem far removed from nature. Yet, nature disperses her own, less conspicuously but with far more bountiful abundance than Juno. Coronaviruses, common viruses of animals and humans, are named for their crownlike appearance. Recently, they came under the spotlight, when an obscure animal coronavirus left its wildlife reservoir to cause SARS, a lethal disease in humans. Nature’s gift that keeps on giving, these viruses continue to emerge, in more species, more places, and now perhaps in North American bats, which could become involved in future emergence in humans or other animals (*8*).
